# Mineral and heavy metal content in dry dog foods with different main animal components

**DOI:** 10.1038/s41598-023-33224-w

**Published:** 2023-04-13

**Authors:** Jagoda Kępińska-Pacelik, Wioletta Biel, Robert Witkowicz, Cezary Podsiadło

**Affiliations:** 1 Department of Monogastric Animal Sciences, Division of Animal Nutrition and Food, West Pomeranian University of Technology in Szczecin, Klemensa Janickiego 29, 71-270 Szczecin, Poland; 2 Department of Agroecology and Crop Production, University of Agriculture in Krakow, Mickiewicza 21, 31-120 Krakow, Poland; 3 Department of Agroengineering, West Pomeranian University of Technology in Szczecin, Juliusza Słowackiego 17, 71-434 Szczecin, Poland

**Keywords:** Zoology, Mass spectrometry

## Abstract

Dog caregivers, mainly for economic reasons and easy availability, choose dry, over the counter diets (OTC). The mineral composition of OTC foods depends primarily on the components used in the production of the pet food. Regardless of the main component of the food, it must meet the recommended minimum mineral content, established by nutritional guidelines. Therefore, the aim of this study was to determine the mineral (Ca, K, Mg, Na, Fe, Mn, Zn, Cu, Mo) and heavy metal content (Pb, Co, Cd, Cr, Ni) using the methods of colorimetry and mass spectrometry, of OTC dry dog foods and to compare with the FEDIAF and AAFCO nutritional guidelines. Dry foods pose no risk to dogs in terms of heavy metal content. The worst results in terms of mineral content were obtained in mixed foods, therefore it is worth considering feeding the dog a mono-protein food. The PCA analysis disproved our hypothesis and revealed that the main animal source did not statistically significantly affect the levels of minerals and their ratios. However, the analysis of contrasts confirms the differentiation of the content of individual minerals between the groups of foods. For the first time, we proved that pet food with a mineral composition similar to the MIN-RL may be characterized by unfavorable mineral ratios.

## Introduction

Dogs population in households is increasing year by year^1^. Dog caregivers, mainly for economic reasons and due to the convenience of feeding and easy availability, choose dry, over the counter diets (OTC). Currently, the pet food market offers a wide range of these products—it is easy to choose the right variant that suits the needs and preferences of the dog. For dog food manufacturers, it should be an important aspect to monitor the content of not only essential nutrients, but also the levels of minerals and heavy metals in the products. Minerals exert important functions in the maintenance of homeostasis, and their excess or deficiency can impair animals’ health^2,3^. Regardless of the main component of the food, it must meet the recommended minimum mineral content. However, viewed as a whole, not only the contents of the individual minerals are important, but also their ratios. The mutual proportions of minerals are necessary for the proper functioning of the body, for the synthesis of enzymes, metabolic changes, etc. Moreover, the presence of heavy metals in the pet food is possible, which depends mainly on the component, its source, but also on the production process of the pet food^4^. Thus, the mineral content of dog food needs to be frequently monitored^5^. Long-term use of the same diet results in correlations between the concentrations of the elements in the blood and in the hair coat^6^. Dark hair contains more Ca and Mg compared to fair hair. In females, higher blood levels of Zn and Mn are observed in comparison to males. Dogs fed raw diets have higher Zn and Se concentrations compared to those fed dry diets. In case of heavy metals, venison consumption is associated with higher blood Pb levels, and blood Pb levels decline as the dog ages^6^. According to the literature data, fish are particularly exposed to the presence of heavy metals^7^. What is important, fish and fish wastes are used to produce pet food^8^. Pet foods containing them are popular in the nutrition of dogs, especially those with food allergies. Poultry is a source of easily digestible protein, but free-range meat can be also contaminated with heavy metals. Beef is a valuable source of iron, and lamb—of copper^9^. Manufacturers should therefore adjust the composition of the product in such a way that the content of both macrominerals and trace elements meets the dog’s needs for these ingredients, while not carrying the risk associated with a deficiency or excess of a given mineral. There are nutritional guidelines in the world that are recommended in Europe^10^ and those that are recommended in the America^11^. Although they refer to the same product as pet food, they differ in the acceptable minimum and maximum levels of some minerals, which may have an impact on the food manufacturers’ practices. We supposed that the mineral composition of OTC foods depends mainly on the components used in the production of the pet food. Therefore, the aim of this study was to determine the mineral and heavy metal content of OTC dry dog foods with different main animal components and to compare mineral adequacy with the FEDIAF^10^ and AAFCO^11^ nutritional guidelines for healthy adult dogs. For this purpose, an analysis of 15 minerals was undertaken, including macrominerals, trace elements and heavy metals.

## Results

### Macrominerals

Among the analyzed dog foods there were 13 foods with fish (F) as the main animal component, 6 with poultry (P), 6 with lamb (L), 6 with beef (B), 6 with at least two animal components (MIX) and a group of 4 other foods (O), one each with deer, ostrich, kangaroo, insects. The FEDIAF and AAFCO nutritional guidelines differ most notably in setting the minimum recommended level (MIN-RL), maximum nutritional level (MAX-RL) for macrominerals and/or legal limit (MAX-LL) for trace elements. For calcium (Ca), both FEDIAF and AAFCO give a recommended MIN-RL—0.5 and MAX-RL—2.5 g/100 g of dry matter (DM) of dog food. Our research showed that the calcium level in 68 percent (F2, F3, F4, F7, F8, F9, F10, F11, F12, F13, P1, P2, P3, P4, P6, L1, B1, B2, B3, B4, B5, B6, M1, M2, M4, M5, O1, O2, O3) of foods was below the MIN-RL (Table 1). The highest statistically significant average level of calcium was obtained for the group of foods with lamb (0.62 g/100 g DM) (Table 2). It was the only food group where nearly all of the foods (except L1) met the MIN-RL of calcium. The groups of foods differed statistically significantly from one another in terms of Ca content, except for the group of foods with poultry (P) and other foods (O). Disturbingly low calcium content was found in M4 food (0.06 g/100 g DM) and the highest—in food L3 (0.75 g/100 g DM). None of the foods exceeded MAX-RL. In terms of phosphorus (P) content, both FEDIAF and AAFCO give the same MIN-RL and MAX-RL (0.4 and 1.6 g/100 g DM, respectively). Statistically significantly the highest mean content of phosphorus was obtained in the F8 food (1.95 g/100 g DM), and its lowest level was found in the B1 food (0.66 g/100 g DM). Significantly the highest mean content of phosphorus was obtained in the group of foods with lamb (1.36 g/100 g DM), while the lowest—in the group of foods with beef (1.07 g/100 g DM). All groups differed statistically significantly in terms of phosphorus content, except for the group of mixed (M) and other foods (O). None of the analyzed foods had phosphorus content below the MIN-RL, while 6 dog foods exceeded the MAX-RL (F8, F9, F13, L2, M6, O1), respectively by 21.25, 7.5, 0.63, 0.63, 5.00 and 9.38 percent. The minimum recommended level for potassium established by FEDIAF is 0.5 g/100 g DM and by AAFCO 0.6 g/100 g DM. If the FEDIAF guidelines were taken into account, only one pet food (L1) did not meet the MIN-RL, while for AAFCO it would be 7 percent of analyzed pet foods (P6, L1, M3). In the case of potassium (K), the highest level was statistically significantly highest in the group of other foods (0.97 g/100 g DM), while the lowest—in the group of foods with lamb (0.74 g/100 g DM). All groups differed statistically significantly in terms of potassium content, except for the group of mixed (M) and foods with beef (B). For magnesium and sodium, all foods analyzed met the recommended minimum levels by both FEDIAF and AAFCO—0.07 and 0.06 g/100 g DM for magnesium, and 0.1 and 0.8 g/100 g DM for sodium. In the case of sodium, significant differences between the groups were found for all groups, except for the foods with poultry (P) and beef (B). The groups of foods also differed statistically in terms of magnesium content. In this case, only no differences were observed between the groups of food with poultry (P) and other foods (O).

**Table 1:** Content of macrominerals (g/100 g DM) in analyzed dry dog foods.

Item	DM	Ca	P	K	Mg	Na
F1	95.44	0.5008^p^	0.8944^b^	1.2714^o^	0.2024^no^	0.6377^w^
F2	91.49	0.3527^cd^	0.9979^cde^	0.8713^ghijklmn^	0.2005^mn^	0.6525^wx^
F3	94.79	0.4900^nop^	1.1814^ijk^	0.8644^ghijklmn^	0.2556^s^	0.5802^uv^
F4	94.37	0.4129^hi^	1.0910^fg^	0.9709^mn^	0.1396^hi^	0.5374^qr^
F5	93.83	0.5987^rs^	1.5076^q^	0.9191^hijklmn^	0.1605^j^	0.7150^y^
F6	94.27	0.4525^jkl^	1.3606^n^	0.9135^hijklmn^	0.2345^r^	0.4252^ijk^
F7	95.05	0.4840^nop^	1.2175^kl^	1.1691^o^	0.2775^t^	1.1017^a^
F8	84.78	0.3903^fg^	1.9462^v^	0.7115^def^	0.0956^cd^	0.4919^n^
F9	93.81	0.4953^op^	1.7260^tu^	0.9578^mn^	0.2122^p^	0.5693^tu^
F10	92.45	0.4340^ij^	1.4207^op^	0.9828^n^	0.2230^q^	0.6643^x^
F11	94.16	0.3262^b^	1.1565^hij^	0.8000^efghijk^	0.1007^d^	0.3507^c^
F12	95.37	0.4975^p^	1.3772^no^	0.8051^efghijk^	0.4374^w^	0.3796^de^
F13	94.78	0.4874^nop^	1.6155^r^	0.9232^ijklmn^	0.2089^op^	0.5405^rs^
P1	92.29	0.4051^gh^	1.2420^lm^	0.6907^cde^	0.1336^gh^	0.3229^b^
P2	95.74	0.3750^ef^	1.2960^m^	0.7903^efghi^	0.0963^cd^	0.3964^f^
P3	92.62	0.4890^nop^	1.1758^ijk^	0.7851^efgh^	0.1747^k^	0.5557^st^
P4	94.41	0.4076^gh^	1.2141^jkl^	0.8053^efghijk^	0.1669^j^	0.4427^l^
P5	84.54	0.6300^t^	1.4859^q^	0.9766^mn^	0.1342^gh^	0.4168^hij^
P6	95.13	0.3861^fg^	1.4269^op^	0.5626^bc^	0.2150^p^	0.3978^fg^
L1	93.07	0.4023^gh^	1.2174^kl^	0.4012^a^	0.0685^a^	0.3685^d^
L2	93.45	0.6794^u^	1.6056^r^	0.7680^defg^	0.3282^u^	0.5223^pq^
L3	95.03	0.7500^v^	1.1460^ghi^	0.7676^defg^	0.3436^v^	0.4131^ghi^
L4	93.09	0.5844^r^	1.4937^q^	0.9289^jklmn^	0.2399^r^	0.4888^n^
L5	93.20	0.6129^st^	1.6429^rs^	0.6446^bcd^	0.1750^k^	0.4309^jkl^
L6	92.83	0.6929^u^	1.0451^ef^	0.9240^ijklmn^	0.2126^p^	0.4352^kl^
B1	94.03	0.4499^jkl^	0.6575^a^	0.9728^mn^	0.1342^gh^	0.3873^ef^
B2	92.08	0.4596^klm^	0.9438^bc^	0.9024^ghijklmn^	0.1885^l^	0.4599^m^
B3	93.61	0.4137^hi^	1.4783^pq^	0.9443^lmn^	0.1084^e^	0.5242^pq^
B4	94.14	0.3738^def^	1.1893^ijkl^	0.8711^ghijklmn^	0.0913^c^	0.4149^hi^
B5	92.59	0.4682^lmn^	1.0954^fg^	0.6338^bcd^	0.1296^g^	0.5039^no^
B6	92.88	0.4811^mnop^	1.0304^de^	0.7925^efghij^	0.1452^i^	0.2583^a^
M1	91.47	0.4493^jkl^	1.2916^m^	0.8081^efghijkl^	0.2021^mno^	0.5564^st^
M2	88.84	0.3372^b^	1.0921^fg^	0.9564^mn^	0.0748^ab^	0.4019^fgh^
M3	92.04	0.6267^t^	1.4270^op^	0.5352^ab^	0.1951^lm^	0.4169^hij^
M4	91.27	0.0580^a^	1.0485^ef^	0.8661^fghijklm^	0.1799^k^	0.2710^a^
M5	92.66	0.4892^nop^	1.1112^gh^	1.2185^o^	0.1285^g^	0.5927^v^
M6	85.66	0.5878^r^	1.6848^st^	0.8442^fghijklm^	0.1780^k^	0.5113^op^
O1 (DEER)	92.19	0.4754^mno^	1.7525^u^	0.9357^klmn^	0.1207^f^	0.3370^bc^
O2 (OSTRICH)	97.44	0.3596^de^	1.1334^ghi^	0.9768^mn^	0.0767^b^	0.8678^z^
O3 (KANGAROO)	95.69	0.4381^jk^	1.1840^ijkl^	1.1560^o^	0.0723^ab^	1.180^b^
O4 (INSECTS)	93.90	0.5261^q^	0.9864^cd^	0.8125^efghijkl^	0.3460^v^	0.3466^c^
MIN-RL^10^		0.50	0.40	0.50	0.07	0.10
MAX-RL^10^		2.50	1.60	*	*	*
MIN-RL^11^		0.5	0.4	0.6	0.06	0.08
MAX-RL^11^		2.5	1.6	*	*	*

DM, dry matter; MIN-RL, minimum recommended level; MAX-RL, nutritional maximum limit; F, foods with fish; P, foods with poultry; L, foods with lamb; B, foods with beef; M, mixed foods; O, other foods; means with at least one same letter in the superscript (a, b, c, …) not differ statistically at *P* = 0.05 (for all columns separately).

* No data.

**Table 2:** Comparison of the means determined for the analyzed groups of dry dog foods based on linear contrasts.

Contrasts Ca					
Item	F ($\overline{x }=0.4556)$	P	L	B	M
P ($\overline{x }=0.4488)$	$\alpha < 0.000$	–	–	–	–
L ($\overline{x} = 0.6203)$	$\alpha < 0.000$	$\alpha < 0.000$	–	–	–
B ($\overline{x} = 0.4411)$	$\alpha < 0.000$	$\alpha = 0.001$	$\alpha < 0.000$	–	–
M ($\overline{x} = 0.4247)$	$\alpha < 0.000$	$\alpha < 0.000$	$\alpha < 0.000$	$\alpha < 0.000$	–
O ($\overline{x} = 0.4498)$	$\alpha = 0.010$	$\alpha =0.690$	$\alpha <0.000$	$\alpha =0.001$	$\alpha <0.000$

Contrasts P					
Item	F ($\overline{x }=1.3456)$	P	L	B	M
P ($\overline{x }=1.3068)$	$\alpha <0.000$	–	–	–	–
L ($\overline{x }=1.3584)$	$\alpha =0.012$	$\alpha <0.000$	–	–	–
B ($\overline{x }=1.0657)$	$\alpha <0.000$	$\alpha <0.000$	$\alpha <0.000$	–	–
M ($\overline{x }=1.2759)$	$\alpha <0.000$	$\alpha <0.000$	$\alpha <0.000$	$\alpha <0.000$	–
O ($\overline{x }=1.2641)$	$\alpha <0.000$	$\alpha <0.000$	$\alpha <0.000$	$\alpha <0.000$	$\alpha =0.071$

Contrasts K					
Item	F ($\overline{x }=0.9354)$	P	L	B	M
P ($\overline{x }=0.7684)$	$\alpha <0.000$	–	–	–	–
L ($\overline{x }=0.7390)$	$\alpha <0.000$	$\alpha =0.033$	–	–	–
B ($\overline{x }=0.8528)$	$\alpha <0.000$	$\alpha <0.000$	$\alpha <0.000$	–	–
M ($\overline{x }=0.8714)$	$\alpha <0.000$	$\alpha <0.000$	$\alpha <0.000$	$\alpha =0.169$	–
O ($\overline{x }=0.9702)$	$\alpha =0.012$	$\alpha <0.000$	$\alpha <0.000$	$\alpha <0.000$	$\alpha <0.000$

Contrasts Na					
Item	F ($\overline{x }=0.5881)$	P	L	B	M
P ($\overline{x }=0.4221)$	$\alpha <0.000$	–	–	–	–
L ($\overline{x }=0.4431)$	$\alpha <0.000$	$\alpha <0.000$	–	–	–
B ($\overline{x }=0.4248)$	$\alpha <0.000$	$\alpha =0.086$	$\alpha <0.000$	–	–
M ($\overline{x }=0.4583)$	$\alpha <0.000$	$\alpha <0.000$	$\alpha <0.000$	$\alpha <0.000$	–
O ($\overline{x }=0.6828)$	$\alpha <0.000$	$\alpha <0.000$	$\alpha <0.000$	$\alpha <0.000$	$\alpha <0.000$

Contrasts Mg					
Item	F ($\overline{x }=0.1807)$	P	L	B	M
P ($\overline{x }=1.5344)$	$\alpha <0.000$	–	–	–	–
L ($\overline{x }=2.2799)$	$\alpha <0.000$	$\alpha <0.000$	–	–	–
B ($\overline{x }=1.3287)$	$\alpha <0.000$	$\alpha <0.000$	$\alpha <0.000$	–	–
M ($\overline{x }=1.5973)$	$\alpha <0.000$	$\alpha <0.000$	$\alpha <0.000$	$\alpha <0.000$	–
O ($\overline{x }=1.5393)$	$\alpha <0.000$	$\alpha =0.531$	$\alpha <0.000$	$\alpha <0.000$	$\alpha <0.000$

F, foods with fish; P, foods with poultry; L, foods with lamb; B, foods with beef; M, mixed foods; O, other foods.

### Trace elements and heavy metals

FEDIAF provides both the MIN-RL (3.60 g/100 g DM) and the MAX-LL (68.18 mg/100 g DM) of iron (Fe), while AAFCO only recommends the minimum level (4.0 mg/100 g DM) (Table 3). All analyzed foods met the MIN-RL for iron, without exceeding the legal limit (MAX-LL). Statistically the highest amounts of iron were found in the M4 food (46.62 mg/100 g DM), while the lowest was determined in food with fish (in the F8 food—4.82 mg/100 g DM). Statistically the highest amounts of iron were found in the group of mixed foods (23.67 mg/100 g DM) (Table 4), while the lowest—in the group of other foods (11.93 mg/100 g DM). All groups of foods differed significantly in terms of iron content. In the case of recommendations regarding the level of zinc (Zn), a similar situation can be observed—FEDIAF provides the MIN-RL (7.20 mg/100 g of DM) and the MAX-LL (22.70 mg/100 g of DM), while AAFCO—only the minimum recommended level (8.0 mg/100 g of DM). The lowest level of zinc was found in the O3 food (2.53 mg/100 g DM). The significantly lowest average zinc level was found in the group of other foods (5.53 mg/100 g DM), and the highest—in the group of foods with fish (14.10 mg/100 g DM). All groups of foods differed significantly in terms of zinc content. Fourteen foods did not meet the FEDIAF and AAFCO minimum recommended levels of zinc, and two foods (F1 and M2) exceeded the legal limit of this trace element (78.95 and 24.92 mg/100 g DM, respectively). The recommended minimum content of manganese (Mn) by FEDIAF is 0.58 mg/100 g DM, and by AAFCO—0.50 mg/100 g DM. The legal limit is specified only by FEDIAF—17.00 mg/100 g DM. Two of the analyzed foods did not meet the recommended minimum level (F8—0.45 and M2—0.54 mg/100 g DM). The highest level of Mn was found in the O4 food (7.56 mg/100 g DM). All groups of foods differed significantly in terms of manganese content. In the case of copper (Cu), the minimum recommended level in dog food is 0.72 and 0.73 mg/100 g DM according to FEDIAF and AAFCO, respectively. The legal limit is 2.80 mg/100 g DM. The significantly highest average content of copper was found in the group of foods with beef (1.84 mg/100 g DM), while the lowest—in the group of other foods (0.88 mg/100 g DM). All groups of foods differed statistically significantly in terms of copper content. Some of the foods did not meet the minimum recommended levels—F8 (0.22 mg/100 g DM), O2 (0.24 mg/100 g DM and O3 (0.16 mg/100 g DM). One food (B1) exceeded the MAX-LL (2.94 mg/100 g DM). The nutritional guidelines do not state limits for molybdenum (Mo), however, its presence was found in all foods at an average level of 0.10–0.11 mg/100 g DM. The analyzes did not detect the presence of cobalt (Co), cadmium (Cd), chromium (Cr) and nickel (Ni) in the foods, while the presence of lead (Pb) was found in five foods (Table 3)—one with fish, two with poultry and two mixed (F1, P1, P2, M1, M5).

**Table 3:** Content of trace elements and heavy metals (mg/100 g DM) in analyzed dry dog foods.

Item	Fe	Zn	Mn	Cu	Mo	Pb
F1	21.0478^j^	78.9548^s^	3.4985^opq^	2.3842^u^	0.0939^def^	0.0462
F2	5.3897^a^	9.5114^j^	1.2269^d^	1.6281^pq^	0.0896^d^	ND
F3	16.2285^f^	12.7166^o^	3.1934^m^	1.6853^q^	0.1037^jkl^	ND
F4	16.7198^f^	9.4209^j^	3.2330^mn^	1.3474^jk^	0.0902^d^	ND
F5	18.4488^hi^	7.9676^hi^	1.6264^f^	0.8894^ef^	0.1043^klm^	ND
F6	33.8628^r^	5.3283^d^	5.2530^w^	1.1722^hi^	0.0908^de^	ND
F7	14.6844^e^	14.3725^q^	4.6591^v^	2.0889^t^	0.1197^r^	ND
F8	4.8184^a^	3.5592^b^	0.4459^a^	0.2170^ab^	0.1465^u^	ND
F9	17.1320^fg^	12.4203^no^	3.2257^mn^	1.1699^hi^	0.1198^r^	ND
F10	18.9084^i^	9.2450^j^	3.2915^n^	1.9156^s^	0.1137^opq^	ND
F11	11.2059^c^	5.6951^de^	0.9771^b^	0.9670^g^	0.1105^no^	ND
F12	31.6787^q^	5.5279^de^	4.6723^v^	1.1838^hi^	0.1129^op^	ND
F13	19.9123^i^	8.5546^i^	3.4026^o^	0.8013^cd^	0.1775^w^	ND
P1	9.8196^b^	12.2435^no^	1.0559^bc^	1.1995^i^	0.1075^lmn^	0.0145
P2	16.6226^f^	8.4077^i^	1.4633^e^	1.3511^k^	0.0943^defg^	0.0172
P3	22.3731^k^	9.2669^j^	2.2490^ij^	0.9604^fg^	0.1004^hijk^	ND
P4	23.3604^lm^	8.0124^hi^	2.8302^l^	1.0073^g^	0.0834^c^	ND
P5	18.6308^i^	11.4502^lm^	0.9871^b^	1.1095^h^	0.0954^efgh^	ND
P6	10.1871^b^	7.6795^gh^	2.2222^g^	1.1642^hi^	0.1049^klm^	ND
L1	13.5232^d^	7.3128^fg^	2.7522^l^	1.4354^no^	0.1067^lmn^	ND
L2	22.5126^kl^	10.9165^kl^	3.4409^op^	1.3703^kl^	0.0916^de^	ND
L3	253578^o^	13.6162^p^	3.7793^r^	1.8036^r^	0.0972^fghi^	ND
L4	23.6279^m^	6.0461^e^	3.8503^rs^	0.9916^g^	0.1180^pqr^	ND
L5	16.8466^fg^	11.8525^mn^	4.0225^t^	1.5306^n^	0.1186^qr^	ND
L6	23.9880^mn^	4.6719^c^	3.5624^q^	1.8313^r^	0.1284^s^	ND
B1	25.3409^o^	10.7689^k^	3.9307^st^	2.9358^v^	0.0928^def^	ND
B2	12.2136^c^	5.4637^de^	5.3356^w^	0.8558^de^	0.1037^jkl^	ND
B3	17.6653^gh^	8.4387^i^	3.9328^st^	1.8022^r^	0.1006^ijk^	ND
B4	11.2670^c^	7.9784^hi^	2.3848^hi^	2.1527^t^	0.1093^mno^	ND
B5	13.9465^de^	9.5588^j^	1.0876^c^	1.1853^i^	0.1048^klm^	ND
B6	24.0397^mn^	5.3128^cd^	4.4590^u^	2.1129^t^	0.1187^qr^	ND
M1	9.6994^b^	10.3843^k^	2.5320^jk^	1.3551^k^	0.1012^ijk^	0.0149
M2	24.5576^no^	24.9195^r^	0.5369^a^	1.1768^hi^	0.0737^b^	ND
M3	16.5445f.	5.2483^cd^	2.2257^g^	0.7285^c^	0.1144^opq^	ND
M4	46.6167^s^	5.5643^de^	2.6219^k^	0.8442^de^	0.0991^ghij^	ND
M5	17.0478^fg^	10.6033^k^	3.5215^pq^	1.2762^j^	0.0656^a^	0.0120
M6	27.5532^p^	7.1755^fg^	3.1491^m^	1.4441^lm^	0.1524^v^	ND
O1 (DEER)	11.8185^c^	6.8039^f^	2.3338^h^	1.4994^mn^	0.1012^ijk^	ND
O2 (OSTRICH)	9.7558^b^	3.0439^ab^	1.0181^bc^	0.2448^b^	0.1107^no^	ND
O3 (KANGAROO)	9.9242^b^	2.5294^a^	1.0842^c^	0.1609^a^	0.1339^t^	ND
O4 (INSECTS)	16.2235^f^	9.7322^j^	7.5563^x^	1.6082^op^	0.0945^fghi^	ND
MIN-RL^10^	3.60	7.20	0.58	0.72	*	*
MAX-LL^10^	68.18	22.70	17.00	2.80	*	*
MIN-RL^11^	4.00	8.00	0.50	0.73	*	*
MAX-RL^11^	*	*	*	*	*	*

DM, dry matter; MIN-RL, minimum recommended level; MAX-LL, legal limit; ND, not detected; F, foods with fish; P, foods with poultry; L, foods with lamb; B, foods with beef; M, mixed foods; O, other foods; means with at least one same letter in the superscript (a, b, c, …) not differ statistically at *P* = 0.05 (for all columns separately).

* No data.

**Table 4:** Contrasts between trace elements in the analyzed groups of dog food.

Contrasts Cu					
Item	F ($\overline{x }=1.3423)$	P	L	B	M
P ($\overline{x }=1.1320)$	$\alpha <0.000$	–	–	–	–
L ($\overline{x }=1.5105)$	$\alpha <0.000$	$\alpha <0.000$	–	–	–
B ($\overline{x }=1.8408)$	$\alpha <0.000$	$\alpha <0.000$	$\alpha <0.000$	–	–
M ($\overline{x }=1.1375)$	$\alpha <0.000$	$\alpha =0.453$	$\alpha <0.000$	$\alpha <0.000$	–
O ($\overline{x }=0.8782)$	$\alpha <0.000$	$\alpha <0.000$	$\alpha <0.000$	$\alpha <0.000$	$\alpha <0.000$

Contrasts Fe					
Item	F ($\overline{x }=17.6182)$	P	L	B	M
P ($\overline{x }=16.8323)$	$\alpha <0.000$	–	–	–	–
L ($\overline{x }=20.9760)$	$\alpha <0.000$	$\alpha <0.000$	–	–	–
B ($\overline{x }=17.2455)$	$\alpha <0.000$	$\alpha <0.000$	$\alpha <0.000$	–	–
M ($\overline{x }=23.6698)$	$\alpha <0.000$	$\alpha <0.000$	$\alpha <0.000$	$\alpha <0.000$	–
O ($\overline{x }=11.9305)$	$\alpha <0.000$	$\alpha <0.000$	$\alpha <0.000$	$\alpha <0.000$	$\alpha <0.000$

Contrasts Mn					
Item	F ($\overline{x }=2.9773)$	P	L	B	M
P ($\overline{x }=1.8330)$	$\alpha <0.000$	–	–	–	–
L ($\overline{x }=3.5679)$	$\alpha <0.000$	$\alpha <0.000$	–	–	–
B ($\overline{x }=3.5217)$	$\alpha <0.000$	$\alpha <0.000$	$\alpha <0.000$	–	–
M ($\overline{x }=2.4312)$	$\alpha <0.000$	$\alpha <0.000$	$\alpha <0.000$	$\alpha <0.000$	–
O ($\overline{x }=2.9981)$	$\alpha =0.032$	$\alpha <0.000$	$\alpha <0.000$	$\alpha <0.000$	$\alpha <0.000$

Contrasts Zn					
Item	F ($\overline{x }=14.0980)$	P	L	B	M
P ($\overline{x }=9.5100)$	$\alpha <0.000$	–	–	–	–
L ($\overline{x }=9.0693)$	$\alpha <0.000$	$\alpha <0.000$	–	–	–
B ($\overline{x }=7.9202)$	$\alpha <0.000$	$\alpha <0.000$	$\alpha <0.000$	–	–
M ($\overline{x }=10.6492)$	$\alpha <0.000$	$\alpha <0.000$	$\alpha <0.000$	$\alpha <0.000$	–
O ($\overline{x }=5.5273)$	$\alpha <0.000$	$\alpha <0.000$	$\alpha <0.000$	$\alpha <0.000$	$\alpha <0.000$

Contrasts Mo					
Item	F ($\overline{x }=0.1133)$	P	L	B	M
P ($\overline{x }=0.0977)$	$\alpha <0.000$	–	–	–	–
L ($\overline{x }=0.1101)$	$\alpha <0.000$	$\alpha <0.000$	–	–	–
B ($\overline{x }=0.1050)$	$\alpha <0.000$	$\alpha <0.000$	$\alpha <0.000$	–	–
M ($\overline{x }=0.1011)$	$\alpha <0.000$	$\alpha <0.000$	$\alpha <0.000$	$\alpha <0.000$	–
O ($\overline{x }=0.1108)$	$\alpha <0.000$	$\alpha <0.000$	$\alpha =0.197$	$\alpha <0.000$	$\alpha <0.000$

F, foods with fish; P, foods with poultry; L, foods with lamb; B, foods with beef; M, mixed foods; O, other foods.

### Mineral ratios

As a result of the alarmingly low content of calcium in the vast majority of analyzed foods, the ratio of calcium to phosphorus (Ca:P) was disturbed. Both the FEDIAF and AAFCO nutritional guidelines recommend that the ratio should be no less than 1:1 and no more than 2:1. The highest ratio of calcium to phosphorus was found in the B1 food (0.68:1), with the lowest phosphorus content. None of the foods met the MIN-RL (Table 5). The correct ratio of sodium to potassium should be 0.20:1 according to FEDIAF and 0.13:1 according to AAFCO. All foods met the recommended minimum Na to K ratios according to both FEDIAF and AAFCO, and the highest statistically significant Na:K ratio was found in the O3 food (1.02:1). Interestingly, despite the very low calcium content in foods below the recommended minimum in most foods, the ratio of calcium to magnesium in all foods was correct—it met the minimum recommended by FEDIAF (1:0.14) and AAFCO (1:0.12). The proper ratio of copper to zinc, based on the nutritional guidelines, should be at least 1:10.00 for FEDIAF and 1:10.96 for AAFCO. The ratio calculated on the basis of FEDIAF was only met in nine foods (22 percent). If AAFCO guidelines were taken into account, none of the groups met the recommended minimum Cu to Zn ratio. The correct iron to copper ratio based on the nutritional guidelines should be at least 1:0.20 for FEDIAF and 1:0.18 for AAFCO. None of the groups met this minimum.

**Table 5:** Mineral ratios in analyzed dry dog foods.

Item	Ca:P	Na:K	Ca:Mg	Cu:Zn	Fe:Cu
F1	0.56:1	0.50:1	1:0.40	1:33.17	1:0.11
F2	0.35:1	0.75:1	1:0.57	1:5.83	1:0.30
F3	0.42:1	0.67:1	1:0.53	1:7.53	1:0.10
F4	0.38:1	0.56:1	1:0.34	1:6.98	1:0.08
F5	0.40:1	0.78:1	1:0.27	1:8.96	1:0.05
F6	0.33:1	0.47:1	1:0.51	1:4.56	1:0.03
F7	0.39:1	0.94:1	1:0.58	1:6.88	1:0.14
F8	0.20:1	0.69:1	1:0.26	1:16.18	1:0.05
F9	0.29:1	0.59:1	1:0.42	1:10.62	1:0.07
F10	0.30:1	0.67:1	1:0.51	1:4.81	1:0.10
F11	0.28:1	0.44:1	1:0.30	1:5.88	1:0.09
F12	0.36:1	0.47:1	1:0.88	1:4.69	1:0.04
F13	0.30:1	0.59:1	1:0.43	1:10.69	1:0.04
P1	0.33:1	0.46:1	1:0.32	1:10.20	1:0.12
P2	0.29:1	0.51:1	1:0.26	1:6.23	1:0.08
P3	0.42:1	0.71:1	1:0.35	1:9.66	1:0.04
P4	0.34:1	0.54:1	1:0.41	1:7.93	1:0.04
P5	0.42:1	0.43:1	1:0.21	1:10.32	1:0.06
P6	0.27:1	0.71:1	1:0.56	1:6.62	1:0.11
L1	0.33:1	0.93:1	1:0.18	1:4.75	1:0.11
L2	0.42:1	0.68:1	1:0.49	1:7.97	1:0.06
L3	0.65:1	0.53:1	1:0.45	1:7.57	1:0.07
L4	0.39:1	0.53:1	1:0.41	1:6.11	1:0.04
L5	0.37:1	0.67:1	1:0.30	1:7.75	1:0.09
L6	0.66:1	0.48:1	1:0.30	1:2.55	1:0.08
B1	0.68:1	0.40:1	1:0.29	1:3.66	1:0.12
B2	0.49:1	0.51:1	1:0.41	1:6.35	1:0.08
B3	0.28:1	0.55:1	1:0.27	1:4.69	1:0.10
B4	0.31:1	0.48:1	1:0.24	1:3.71	1:0.19
B5	0.43:1	0.79:1	1:0.28	1:8.03	1:0.09
B6	0.47:1	0.33:1	1:0.31	1:2.52	1:0.09
M1	0.35:1	0.69:1	1:0.44	1:7.63	1:0.14
M2	0.31:1	0.42:1	1:0.21	1:21.12	1:0.05
M3	0.44:1	0.78:1	1:0.32	1:7.19	1:0.04
M4	0.06:1	0.31:1	1:3.00	1:6.62	1:0.02
M5	0.44:1	0.48:1	1:0.27	1:8.28	1:0.08
M6	0.35:1	0.61:1	1:0.31	1:4.99	1:0.05
O1 (DEER)	0.27:1	0.36:1	1:0.25	1:4.53	1:0.13
O2 (OSTRICH)	0.32:1	0.89:1	1:0.22	1:12.67	1:0.02
O3 (KANGAROO)	0.37:1	1.02:1	1:0.16	1:15.81	1:0.02
O4 (INSECTS)	0.54:1	0.43:1	1:0.66	1:6.04	1:0.10
MIN-RL^10^	1:1	0.20:1	1:0.14	1:10.00	1:0.20
MAX-RL/LL^10^	2:1	*	*	*	*
MIN-RL^11^	1:1	0.13:1	1:0.12	1:10.96	1:0.18
MAX-RL^11^	2:1	*	*	*	*

MIN-RL, minimum recommended level; MAX-RL/LL, nutritional maximum/legal limit; F, foods with fish; P, foods with poultry; L, foods with lamb; B, foods with beef; M, mixed foods; O, other foods.

* No data.

### Comparative analysis

**Figure 1:**
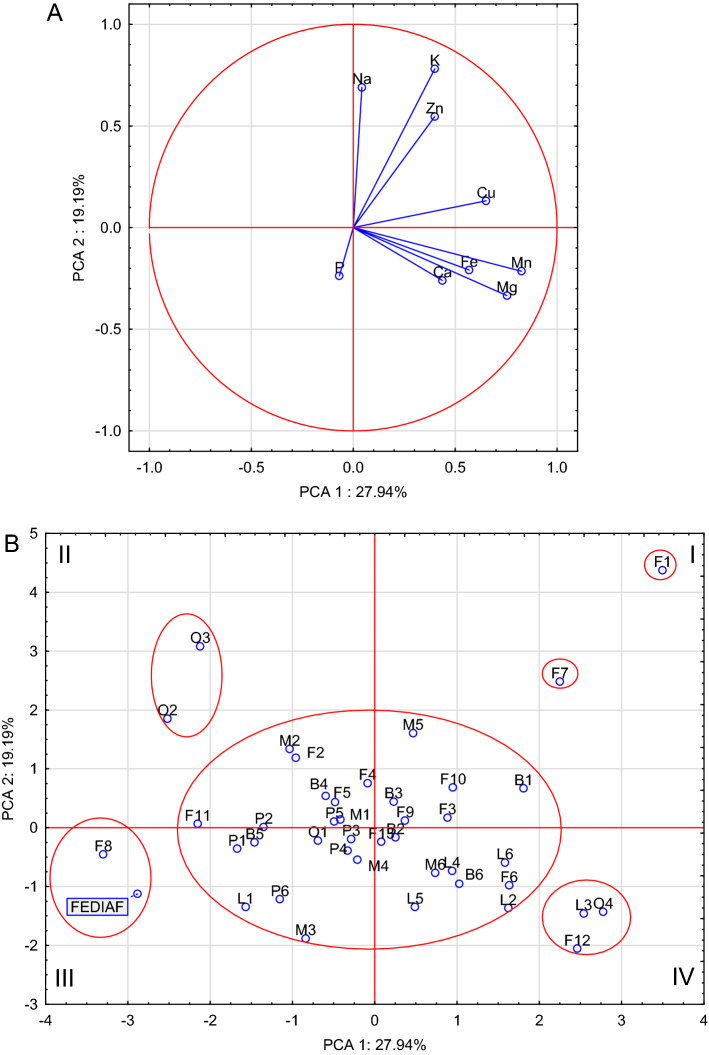
Biplot based on first two principal component axes for mineral composition of analyzed dry dog foods (**A**) and distribution of 41 dry dog foods based on the first two components obtained from principal component analysis (**B**).

**Figure 2:**
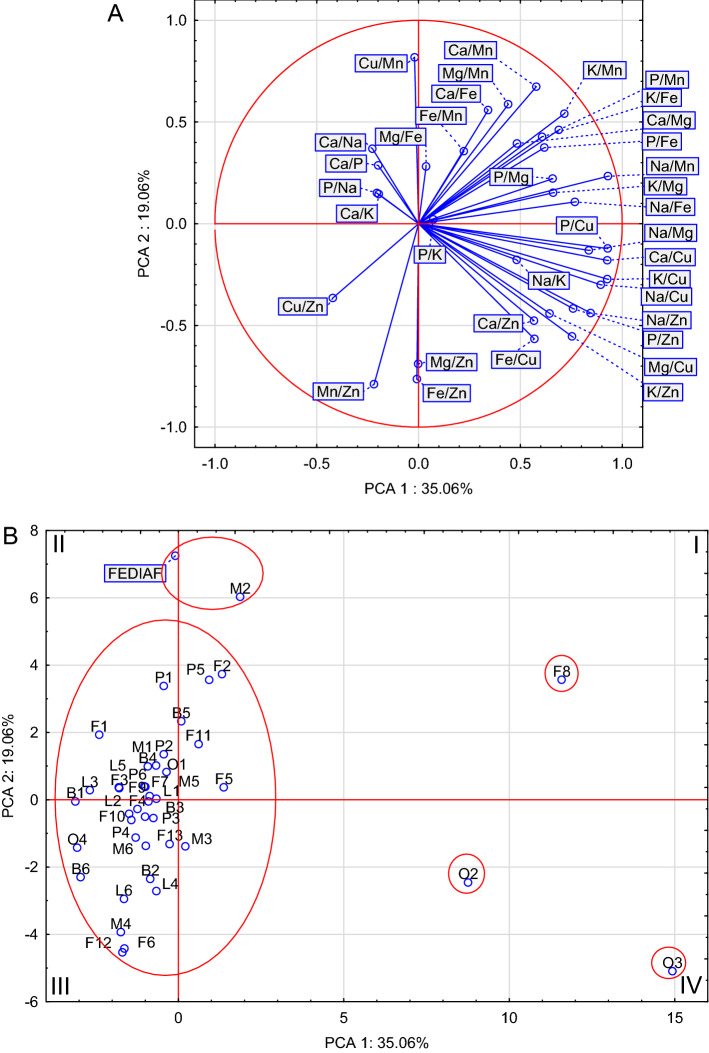
Biplot based on first two principal component axes for mineral ratios of analyzed dry dog foods (**A**) and distribution of 41 dry dog foods based on the first two components obtained from principal component analysis (**B**).

PCA analysis based on the mineral composition of the dry dog foods indicated that the first two components accounted for 47.13 percent of the total variance (Fig. 1A). The analysis allowed the distribution of the foods in the quadrants of the 2 factor coordinate plot for the cases (Fig. 1B). The foods that make up single-element sets in quadrant I are F1 and F7. These foods are distinguished by a particularly high content of K and Zn, but also all foods in this quarter were characterized by an increased content of Cu and Na. Quarter IV foods contain a higher content of Mn, Mg, Fe and Ca than other foods. Foods forming a separate group in this quarter, with a particularly high content of the above-mentioned elements, are foods F12, O4 and L3. It is worth emphasizing that this quarter contains the vast majority of foods with lamb. Quarter III includes the FEDIAF^10^ guidelines and forms a group with F8 food, which suggests a close but not complete compliance of its mineral composition with the MIN-RL, which confirms that it does not meet the requirements for Mn and Cu. Generally, foods in this quarter have a higher content of phosphorus than in other foods. In quarter II, a separate group is formed by O2 and O3 foods, which have in common the low content of minerals, especially Mn, Mg, Fe and Ca (Fig. 1A). The PCA analysis carried out on the ratios of elements showed that the source of animal raw materials did not significantly affect the ratios of elements in the tested foods, because in the coordinate system there were no uniform groups of foods determined on the basis of the main component (Fig. 2A, B). For example, foods from the others group, which are in different quarters, such as food O1 (quadrant II) and food O3 (quadrant IV). The ratios of the content of elements in the absolute majority of pet foods are far from those resulting from the FEDIAF. The presence of O3, O2 and F8 foods on the market should be emphasized, the ratios of which were extremely unfavorable, which should raise doubts as to the safety of their long-term administration to animals. These foods were characterized by narrow Ca:P, Ca:Mg, and Fe:Cu ratios and a wide Cu:Zn ratio. The conducted analysis also allows to indicate one food (M2) whose mineral content ratios are close to the ratios resulting from FEDIAF. It is worth emphasizing that the food with a similar composition to MIN-RL was the F8 food, but the food with the most similar ratios was the M2.

## Discussion

A complete and balanced food is essential for the health and well-being of dogs. Adequate diets at every stage of life provide the nutrients needed for reproduction, growth and a long, healthy, active adult life. They also prevent nutritional disorders that can occur due to nutritional deficiencies or excesses. Both the FEDIAF and AAFCO nutritional guidelines establish minimum recommended levels for macrominerals (Ca, P, K, Na, Mg, Cl) and trace elements (Cu, I, Fe, Mn, Se, Zn), however, the FEDIAF guidelines also provide a maximum level of a nutrient in a complete pet food and legal limit for most of them (MAX-RL—Ca, P; MAX-LL—Cu, I, Fe, Mn, Se, Zn). The legal limit applies only when a specific element is included in the recipe as an additive, but it applies to the total amount present in the finished product, and if it comes solely from the ingredient, it does not apply. The legal limit is mandatory and always applies to all life stages. The nutritional maximum is the highest level that is not supposed to cause any harmful effect. Unless the life stage is indicated it applies to all life stages^10^. The mineral content found will be compared to both MAX-RL (macrominerals) and MAX-LL (trace elements), in order to show irregularities in the balance of foods in terms of mineral content. In our research, the level of calcium was disturbing, which in the vast majority of foods was below the MIN-RL. Calcium deficiency may disturb homeostasis and lead to negative health consequences. Calcium deficiency resulting in hypocalcemia may be one of the causes of progressive lethargy, exercise intolerance, hind limb paresis, muscle atrophy and hyperesthesia^12^. Research by Atwal et al.^13^ in adult dogs shown that excess dietary calcium does not lead to adverse health effects, which means that a deficiency in the diet should be of more concern. Secondary hyperparathyroidism (NSHP), osteodystrophy and osteopenia are common complications that can occur in animals consuming an unbalanced diet. The root cause of NSHP is calcium deficiency, which can occur due to an inability to absorb Ca, a lack of dietary Ca and/or vitamin D, or excessive phosphorus intake, even when Ca intake is sufficient^14^. By definition, a complete food should be balanced in such a way as to meet the MIN-RLs for all minerals. However, as our research shows, additional supplementation with calcium-rich products may be necessary in some cases. In a dog’s diet, the sources of calcium can be primarily bones or eggshells. According to the research of Ebeledike et al.^15^ the best source of calcium among animal products is chicken meat and offal, followed by goat, cattle and pig. In our study, dog foods with lamb were best in terms of calcium content, containing 0.62 g/100 g of DM calcium. In the studies of Pereira et al.^16^, the average calcium level in adult dog food was 1.94 g/100 g DM, so it was almost three times higher than the levels found in our studies. Despite this, it was within the recommended levels, not exceeding MAX-RL. Sgorlon et al.^17^ found an average calcium content of extruded dry foods of 1.08 g/100 g DM, which was also higher than the levels determined in our study. Phosphorus has a major role in energy metabolism as a component of adenosine triphosphate^18^. Importantly, the consumption of diets rich in phosphorus is associated with adverse effects on kidney function parameters^19^. This is especially dangerous for dogs suffering from kidney disease. In addition, wet foods have been shown to pose a risk in this regard as they contain much higher levels than the FEDIAF recommended MIN-RL, exceeding the MAX-RL^20^. According to studies^15,21,22^, the content of phosphorus in the meat of individual animal species can be ranked as follows: fish > beef > lamb > poultry > pork. The largest amounts are contained in the bone elements of these animal species^15^. Long-term deficiency of phosphorus in the diet leads to hypophosphatemia, anorexia, disturbances in appetite and growth^23^. Elevated serum phosphate levels are a recognized risk factor for cardiovascular disease and mortality in chronic kidney disease^23^. As shown by Dobenecker et al.^2^ the use of certain inorganic phosphates in pet food is potentially harmful and should be limited. In our research, the highest average level of phosphorus was found in foods with lamb (1.36 g/100 g DM) and fish (1.35 g/100 g DM). The overall average of all foods was 1.28 g/100 g DM. The results found are higher than those obtained by Sgorlon et al.^17^ (1.0 g/100 g DM) and Pereira et al.^16^ (1.17 g/100 g DM). Potassium aids in the functioning of electrical charges in the heart, nerves, and muscles. Sodium aids in the production and transmission of nerve impulses. In dog diet good sources of sodium can be eggs^24^. Meat and fish are good sources as well, but processed ones contain more sodium^25^. This means that dogs fed with “human” food and getting leftovers are exposed to higher sodium doses. In our study, the highest level of potassium was found in group of other foods (0.97 g/100 g DM) and foods with poultry (0.94 g/100 g DM). In the studies of Pereira et al.^16^, an average of 0.77 g/100 g DM potassium was found, and in Sgorlon et al.^17^—0.77 g/100 g DM. In case of sodium, its highest amounts were found in group of other foods (0.68 g/100 g DM) and foods with poultry (0.42 g/100 g DM). The overall average of all foods was 0.50 g/100 g DM. In the studies of Pereira et al.^16^, an average of 0.69 g/100 g DM sodium were found and was higher than in our research. However, Sgorlon et al.^17^ found lower sodium content in dog foods—at the level of 0.32 g/100 g DM. Magnesium is important in many biological functions as a coenzyme in the body of both humans and animals^26^. Due to the proliferative nature of blood cells, the microenvironment which regulates the latency of the hematopoietic stem cells are key to the survival of animals in health and disease^27^. Magnesium disorders have been associated with prolonged hospitalization and higher mortality rates, with neurological and cardiovascular clinical signs^28^. In our study, the highest amount of magnesium was found in foods with lamb (0.23 g/100 g DM). In study by Pereira et al.^16^ magnesium content was determined at level of 0.15 g/100 g DM which was in line with our results for poultry, beef, mix and other foods. Sgorlon et al.^17^ obtained lower results—at an average level of 0.10 g/100 g DM. The nutritional guidelines provide pet food manufacturers nutritional recommendations to ensure the production of a balanced and nutritionally healthy pet food, and is periodically updated based on the latest scientific publications. An example of a change that has occurred to the FEDIAF nutritional guidelines is the legal limit for iron in dry dog food. In the 2019, it was 142 mg/100 g DM for food, while in the latest guidelines it was reduced to 68.18 mg/100 g DM. Because, in dogs, the centrilobular hepatic region is where copper accumulates, colocalization of copper and toxic metabolites or electrophiles magnifies the risk of regional cellular injury. Of additional concern is that centrilobular regions are the last zone to extract oxygen from sinusoidal blood. Consequently, these hepatocytes have a heightened risk for ischemic, hypotensive, or hypoxia-related oxidative injury. Thus, colliding factors may initiate sudden hepatocyte injury in dogs with substantial centrilobular copper accumulation. Examples of this so-called “2-hit phenomenon” include sudden dramatic increases in serum alanine transaminase activity in combination with severe hemolytic or blood loss anemia, hypoxemia, cardiac failure, hypotensive shock, gastric dilatation volvulus, hypotension during general anesthesia, and administration of cytochrome P450-metabolized xenobiotics (e.g., NSAIDs) that form oxidative adducts or electrophiles^29^. The greatest amount of iron in animal products can be found in lamb and beef compared to chicken and pork^9^. The excess iron acts as a poison in dog, and causes damage to the gastrointestinal, liver, metabolic, nervous, and cardiovascular systems. Iron is used by nearly all cells in the body and is an important component of many processes, including erythropoiesis^30^. In veterinary medicine, dietary iron deficiency in adult dogs is rarely seen^31^. In our research, mixed foods were the richest in terms of iron content (23.67 mg/100 g DM). In the studies of Sgorlon et al.^17^ found an average iron level of 16.54 mg/100 g DM, which was similar to the value we found in our research in foods with poultry (16.83 mg/100 g DM). An excess of iron in the diet can lead to mild gastrointestinal damage. High levels of copper interfere with iron absorption, which reduces its use by the body. This can lead to anemia and frequent diarrhea^32^. In our study, the level of copper in all foods was lower than the level of iron, which resulted in low ratios of these elements, similar to the studies of Pereira et al.^16^ and Sgorlon et al.^17^. Copper is involved in erythropoiesis and supports iron absorption. Participates in pigmentation of the skin and hair. Deficiency, although rare, can cause muscle dysfunction. In the liver, it can cause acute hepatitis with immediate consequences, or it can cause chronic damage over time, leading to extensive scarring (cirrhosis) and liver failure. Excessive accumulation of copper in the liver as a cause of hepatitis and cirrhosis was first demonstrated in Bedlington terriers. Copper excess can be found in dogs with normal liver histology, dogs with hepatitis, and dogs with end-stage cirrhosis. A growing body of evidence suggests that copper is a cause of liver disease in non-Bedlington dog breeds^33^. Excess copper causes damage to hepatocytes^29^. Among slaughter animals, the richest source of copper is lamb^9^, therefore it is assumed that lamb foods should contain the most of it, and therefore should be used with caution in the nutrition of dogs with liver problems. Our research does not confirm that foods with lamb are supposed to be a good source of copper. Its highest levels were found in group of foods with beef (1.84 mg/100 g DM) and lamb (1.51 mg/100 g DM), which was similar to the studies by Sgorlon et al.^17^ (1.50 mg/100 g DM). On the other hand, Pereira et al.^16^ found a 70 percent higher average copper level of 2.28 mg/100 g DM. Zinc is important for cellular immunity, reproductive and skin function, and wound healing^34^. The best source of zinc is beef^9^. In our research, the highest average level of zinc was found in the group of foods with fish (14.10 mg/100 g DM), because the F1 food almost four times exceeded the MIN-RL, which influenced the increase in the average obtained in this group of foods. There is a risk that the producer could have used a different raw material for the production, not declared on the label, which resulted in a very high level of zinc in this pet food. The vegetable component in this food was, among others, chickpeas, which are a rich source of zinc. The obtained result may therefore be a reflection of the component composition. It may also be related to the accumulation of zinc in the raw material, depending on the feed consumed by the fish or on the mineral supplements used in the F1 food production. In Sgorlon et al.^17^ study, the average level of this element in extruded dry dog foods was 23.15 mg/100 g DM, while in study by Pereira et al.^16^ 32.50 mg/100 g of DM. In a study by Gregório et al.^34^ the average content of zinc in dry foods was about 29.52 g/100 g DM. Importantly, most of the foods had Zn levels higher than the established limit (22.7 mg /100 g DM). High levels of copper in the diet and low levels of zinc are significantly associated with high levels of copper in the liver. Dietary copper and zinc at current levels in commercially available dry dog food may affect copper in the liver and may be a risk factor for the development of copper-associated hepatitis in Labrador Retrievers with a genetic susceptibility to copper^35^. In our study, the average level of copper in all food groups was ten times lower than that of zinc, as in Pereira et al.^16^ and Sgorlon et al.^17^. Manganese activates the enzymes that are needed to build collagen, which gives strength to soft tissues. According to the research of Gerber et al.^9^ from meat, lamb is the best source of manganese in comparison with foal, chicken, beef, pork. Compared to the literature data, the level of manganese in our study was relatively low. The highest was found in the group of foods with lamb (3.57 mg/100 g DM), and compared to the average results obtained by Pereira et al.^16^ (7.98 mg/100 g DM) and Sgorlon et al.^17^ (10.66 mg/100 g DM) the level was quite low. As a result of natural processes and human activity, molybdenum enters soil and water, and then enters the food chain through plants and livestock. Normally pure molybdenum is not added to pet food, but ordinary ingredients carry this element. Experiments in rats have shown that molybdenum is an essential nutrient, while excessive consumption is toxic^36^. These characteristics of molybdenum most likely apply to dogs and cats. The toxicity of high molybdenum intake animals is related to the induction of secondary copper deficiency; the condition can be cured or prevented with copper supplementation^37^. Among slaughter animals, the best source of molybdenum is skinless chicken breast in comparison with foal, lamb, beef, pork^9^. In our study, the amounts of molybdenum found were relatively similar in all food groups (average 0.100–0.110 mg/100 g DM), but higher than those found by Pereira et al.^16^ (0.049 mg/100 g DM). In order to increase the proportion of minerals in the diet, vitamin-mineral supplements^38^ can be used, which is common practice among dog caregivers on a home or raw diet. Most vitamin and mineral supplements, in the amounts recommended by manufacturers, do not guarantee the minimum nutritional recommendations for the following elements: calcium, potassium, magnesium, sodium, phosphorus, selenium and zinc. In study by Zafalon et al.^38^ most vitamin and mineral supplements have been found to fall short of minimum recommendations for the most important minerals, and when formulated by untrained professionals, even with supplementation, homemade food may still be deficient in nutrients. Moreover, some analyzed vitamin and mineral supplements may suggest a risk of mercury poisoning in domestic animals^38^. Our research did not detect the presence of Co, Cr, Ni. However, they can be present in dog food^16,17^. In the study by Pereira et al.^16^, the content of Co, Cr, Ni in dog foods was at the level of 0.014, 0.076, 0.081 mg/100 g DM, respectively. On the other hand, in study by Sgorlon et al.^17^ the level of these elements was 0.025, 0.153, 0.093 mg/100 g DM. These data indicate that although these elements are present in small amounts in food, dogs are still exposed to them due to the possibility of accumulation. A type of threat are heavy metals, the sources of which in food chains are pollution, soil, machinery and water used in production. The most dangerous heavy metals unnecessary for the body, include cadmium (Cd), lead (Pb), mercury (Hg) and arsenic (As). Their impact on the dog body is varied and depends on the amount of the absorbed dose, duration of the contamination process and the age of the animal. These elements have the highest accumulation factor. They are involved in the development of diseases of the nervous, skeletal and blood systems. They also have a carcinogenic effect, contributing to the development of neoplasms. The maximum allowable levels (MAL) of heavy metals in foodstuffs are regulated by the Commission Regulation (EC) establishing the MAL of certain contaminants in foodstuffs, but there is no regulation specifying the level of these contaminants in pet food. According to Directive 2002/32/EC^39^, referred to in the Code of Good Manufacturing Practice^40^ and the subsequent document amending them^41^, the level of lead in complete feed (i.e. within the meaning of the legal act—also in complete dog food) should not exceed the maximum content of 5 mg/kg in a corresponding feed with a moisture content of 12 percent. Moreover, in the case of food products, in 2021 the EU lowered the limits for heavy metals—cadmium and lead^42,43^. The source of heavy metals can be pet food ingredients (vegetables, fruits, and animal products). Pork fat had almost three times higher concentrations of arsenic, than fish oil and poultry fat. Moreover, pork fat compared to fish oil and poultry fat, was the only one containing mercury^44^. Other studies indicate that foods with fish as the main meat component, which contain higher amounts of heavy metals than foods based on chicken or red meat^4^. Our research analyzed the levels of cadmium and lead. Cadmium was not found in any of the foods, while lead was present in one fish, two poultry and two mixed foods. The highest level found was for food with fish—0.046 mg/100 g DM. In the study of Sgorlon et al.^17^ the average level of cadmium and lead found was 0.030 and 0.307 mg/100 g DM, while in Pereira et al.^16^ 0.015 and 0.018 mg/100 g DM. It has been shown that dry food has a higher concentration of most heavy metals than wet food. Although the amounts found are not high, it should be borne in mind that chronic exposure to their presence has negative health consequences, however, levels of chronic exposure to toxicity are very unlikely. In a study by Kim et al.^4^, fish-based foods had significantly higher arsenic, cadmium, and mercury content than poultry or red meat-based diets. Red meat-based diets (beef, venison, and bison) were characterized by a higher concentration of lead than poultry and fish diets. Based on these results, it can be concluded that commercial dog food appears safe for chronic consumption, and the concentration of heavy metals depends on the primary protein sources^4^. While our research has focused on the mineral content of foods depending on the animal raw material, it is worth bearing in mind that sometimes the composition of the pet food is balanced in such a way that it is impossible to naturally provide all the minerals in amounts that meet the MIN-RL. Therefore, manufacturers use permitted mineral dietary supplements to increase the level of a given mineral in dog food, in the form of sulfates, carbonates and other compounds. However, the safety of such practices is questioned, as underestimating the proportion of “artificial” additives—which will be discussed later—may adversely affect the health of the animal, therefore it is necessary to monitor the composition of foods and establish safe levels of dietary additives, including minerals. An example of a mineral whose differences in bioavailability should be analyzed is ferrous carbonate, which is generally less bioavailable than ferrous sulfate. While iron carbonate may be effective for adult animals, it would be insufficiently bioavailable for young animals which require a highly effective iron source for a rapid response in hemoglobin synthesis^45^. Given the limited information on iron toxicity in dogs and potential differences between breeds in trace element sensitivity, it is imperative to analyze the exact composition of complete feeds not only for adult animals, but also for young ones, which should be the subject of our next research. Because of the close relationship of calcium and phosphorus, AAFCO and FEDIAF guidelines provide a minimum calcium-to-phosphorus ratio of 1:1 and a maximum ratio of 2:1. In our study, the best balanced foods in terms of calcium to phosphorus ratio were foods with lamb, while the worst in this respect were mixed foods, with at least 2 different species as the main animal components. In one mixed food, the ratio was 0.06:1, which is undoubtedly a very disturbing result. In the study of Pereira et al.^16^ the Ca:P ratio was 1.64:1, while in the study by Sgorlon et al.^17^ − 1.1:1, these results do not confirm ours. Importantly, a clinical study found that any growing dog that did not receive the appropriate food and supplements depending on age and breed showed joint thickening, long bone inversion, fore and hind limb inversion, limb unfolding, and difficulty moving^46^. It is important because the caregivers of dogs growing after the age of 1 year often switch from puppy food to maintenance food for adult dogs, despite the fact that dogs of large breeds grow slower and longer need appropriate food for growing dogs, not only with the appropriate proximate composition, but also with an appropriate content of minerals, including the essential calcium and phosphorus, which are involved in the development of the skeleton. A reduced Na:K ratio is a common symptom in patients with adrenal insufficiency, and adrenal insufficiency is considered one of the more likely diagnoses in dogs with a low Na:K ratio^47^. Sgorlon et al.^17^ found potassium and sodium content in dog foods at the level of 0.46 and 0.32 g/100 g DM, respectively, which gives Na:K ratios 0.89:1 and 0.70:1, respectively. In our research, the ratio of 0.70:1 was found in the group of other foods. The lowest ratio was found in the group of foods with beef (0.49:1). The action of magnesium in the body is suppressed by excess calcium, which may contribute to calcium deposition in the urinary tract and gallbladder. Highest ratio of calcium to magnesium may has a certain relationship with the incidence rate of urolithiasis in dog. In our research, the ratio of these elements ranged from 1:0.30 in foods with beef to 1:0.46 in those with fish. In the studies of Pereira et al.^16^, this ratio was 1:0.13, while in Sgorlon et al.^17^ 1:0.10.

An important issue that cannot be overlooked in the context of the mineral composition of complete dog foods is the content of additives. Many additives are used in dog food to increase the taste of the food, extend its freshness or have a positive effect on the animal's body, for example by improving the functioning of joints or enriching the intestinal microflora. All additives must be approved for use and administered in the correct amounts^48^. In the countries belonging to the European Union, the matter of additives used in animal nutrition is regulated by the Regulation of the European Parliament and of the Council^49^. Depending on their properties and functions, additives have been divided into the following categories^49^: (1) technological additives—substances that prolong the freshness of food and are responsible for its structure, including preservatives, antioxidants, emulsifiers, stabilizers; (2) sensory additives—dyes and preparations aimed at improving the taste and smell of food; (3) nutritional additives—positively affecting the functioning of the body, for example vitamins, minerals and amino acids; (4) zootechnical additives—substances that increase digestibility and have a positive effect on the intestinal microflora; (5) coccidiostats and histomonostats—substances limiting and inhibiting the development of protozoa.

Mineral substances added to pet food therefore belong to point 3—nutritional additives. However, adding them can be problematic, because it can lead to their excess in the diet. As shown by Kazimierska et al.^50^, for example, Zn in dog foods was more likely to be in excess than in deficit. Interestingly, dog foods exceeding the maximum legal limit of Zn, Cu and Fe were fortified with nutritional additives, although apparently they did not require any supplementation, as this led to exceeding the maximum legal limit. In the studies of these authors, it was also observed that dog foods without declared supplementation with nutritional additives met the minimum recommended levels of trace elements, and in two foods they even exceeded the acceptable level. Interestingly, according to the Code of Good Labelling Practice for Pet Food, produced by the European Pet Food Industry Federation, there is no obligation to declare additives with no legal maximum limit^48,51^.

Dry foods pose no risk to dogs in terms of heavy metal content. The worst results in term of mineral content were obtained in mixed foods, therefore it is worth considering feeding the dog a mono-protein food. When feeding dogs with extruded dry foods, supplementation with calcium may be necessary due to its deficiency in foods, especially in mixed foods, with components from at least two different types of animals. The PCA analysis disproved our hypothesis and revealed that the main animal source did not statistically significantly affect the levels of minerals and their ratios. What is more, pet food with a mineral composition similar to the MIN-RL may be characterized by unfavorable mineral ratios.

## Methods

### Material

Over the counter compete dry dog foods (41) for adult dogs were selected in packages from the range of 0.3 to 2.5 kg. These foods were randomly chosen at Polish pet stores and online stores. Dog foods were bought in January 2022. Foods were divided into groups depending on the main animal component: fish (F), poultry (P), lamb (L), beef (B). A group of mixed foods (M) was distinguished, which contained at least two different sources of animal components, and a group of other foods (O) with unconventional sources of animal protein (Table S1). All bags were stored in laboratory room temperature (approx. 18–21 °C) until analyzes. Bags were opened on the same day. From each bag, a representative sample was collected for laboratory analyzes^52^. Samples of foods were ground in a laboratory mill type KNIFETEC 1095 (Foss Tecator, Höganäs, Sweden), placed in sterile containers and marked with the symbols 1–41 (Tables 1, 2, 3, 4, 5).

### Chemical analyzes

To determine the dry matter, the samples were dried at 105 °C until constant weight according to AOAC^53^. The total content of potassium (K), calcium (Ca), magnesium (Mg), sodium (Na), iron (Fe), manganese (Mn), zinc (Zn), copper (Cu), molybdenum (Mo), lead (Pb), cobalt (Co), cadmium (Cd), chromium (Cr) and nickel (Ni) was determined by wet mineralization in a mixture of nitric acid (V) and perchloric acid (VII)^52^. The analyzes were performed with an atomic absorption spectrometer (Thermo Fisher Scientific iCE 3000 Series, Waltham, Massachusetts, USA). For the determination of Ca, K, Mg and Na the wavelengths were determined: K: 766.5 nm; Ca: 422.7 nm; Mg: 285.2 nm; Na: 589.0 nm. For the determination of: Fe, Mn, Zn, Cu, Mo, Pb, Co, Cd, Cr, Ni- Fe: 248.3 nm; Mn: 279.5 nm; Zn: 213.9 nm; Cu: 324.8 nm; Mo: 313.3 nm; Pb: 207.2 nm; Co: 240.7 nm; Cd: 228.8 nm; Cr: 357.9 nm; Ni: 232.0 nm. The calculation of the content of the individual elements was started with a standard curve taking into account the weight of the test portion and the dilutions used. The material for P concentration analyses was subjected to mineralization in concentrated sulfuric acid (H_2_SO_4_) and perchloric acid (HClO_4_). The phosphorus (P) content was determined by the Egner-Riehm colorimetric method, with ammonium molybdate on a Specol 221 apparatus spectrophotometer (Carl Zeiss Jena, Germany). The absorbance value of the sample, determined spectrophotometrically, from P_2_O_5_ to total phosphorus, was calculated according to the chemical equivalent (0.436). The credibility of the method used has been confirmed by comparative studies, incl. calibration curve, using the calibration series method. Macrominerals content was expressed as g per 100 g DM of dog food, the content of trace elements and heavy metals was expressed as mg per 100 g DM of dog food. All chemical determinations were performed in triplicate and presented as mean values. The accuracy of the analytical methods was verified based on certified reference material skimmed milk powder (ERM®-BD151), which was obtained from the Institute for Reference Materials and Measurements (IRMM, Geel, Belgium).

### Nutritional adequacy

The results of the analyzes are expressed in 100 g DM (Tables 1 and 3). The demand for nutrients recommended by the nutritional guidelines is expressed in a unit per 100 g DM, assuming an energy density of 4 kcal^10^. The levels of all nutrients were compared to minimum recommended level (MIN-RL), nutritional maximum limit (MAX-RL) and legal limit (MAX-LL)^10,11^. In the case of mineral ratios, the nutritional guidelines provide MIN-RL and MAX-RL for Ca and P only. That is why the mineral ratios were calculated on the basis of the recommended content of a given element in dog food.

### Statistical analyzes

Statistical analyzes were carried out using the STATISTICA v13.3 software (TIBCO Software Inc., Palo Alto, CA, USA)^54^. The significance of differences between the means was assessed using the Tukey test at *P* = 0.05. The method of contrasts was used to compare the means for the groups of dog foods.

## Supplementary Information

Supplementary Information.

## Data Availability

All data generated or analyzed during this study are included in this published article (and its Supplementary Information files).
