# Supplementary material for: Mineral and heavy metal content in dry dog foods with different main animal components
Source: Sci Rep. 2023 Apr 13;13:6082. doi: 10.1038/s41598-023-33224-w (PMC10102197; doi:10.1038/s41598-023-33224-w)
Supplement: Supplementary file 1 — Supplementary Information. [file 41598_2023_33224_MOESM1_ESM.docx]

**Table S1**

Main declared ingredients of analyzed dog foods.

| Item | Main animal ingredients | Main plant ingredients |
| --- | --- | --- |
| F1 | fish | sweet potatoes, lentils, tapioca, chickpeas |
| F2 | fish | oat, lentils, pea, chickpeas |
| F3 | fish | potatoes |
| F4 | fish | sweet potatoes, pea seeds, amaranth |
| F5 | fish | sweet potatoes, potatoes, pea seeds |
| F6 | fish | potatoes |
| F7 | fish | spelt, oat |
| F8 | fish | starch of undetermined origin |
| F9 | fish | linseed, sweet potatoes, pea, potatoes |
| F10 | fish | sweet potatoes, potatoes, pea |
| F11 | fish | sweet potatoes, pea, potatoes |
| F12 | fish | potatoes |
| F13 | fish | sweet potatoes, pea, potatoes |
| P1 | poultry | barley, corn, millet |
| P2 | poultry | potatoes, sweet potato |
| P3 | poultry | potatoes |
| P4 | poultry | potatoes |
| P5 | poultry | sweet potatoes, potatoes |
| P6 | poultry | rice, oats, corn |
| L1 | lamb | rice |
| L2 | lamb | potatoes |
| L3 | lamb | potatoes |
| L4 | lamb | sweet potatoes, pea, potatoes |
| L5 | lamb | rice |
| L6 | lamb | sweet potatoes, pea, potatoes, linseed |
| B1 | beef | potatoes, lentils |
| B2 | beef | rice |
| B3 | beef | sweet potatoes, pea seeds, potatoes, chickpeas |
| B4 | beef | pea starch, tapioca |
| B5 | beef | pea, pea seeds, wheat |
| B6 | beef | sweet potatoes, potatoes, pea, linseed |
| M1 | meat and animal derivatives | cereals |
| M2 | beef, poultry | potatoes |
| M3 | meat and animal by-products | corn |
| M4 | meat and animal by-products | rye, rice |
| M5 | poultry, pork, beef | sweet potatoes, beet pulp, tapioca, lentils, chickpeas |
| M6 | venison, lamb, poultry, rabbit | sweet potatoes |
| O1 | deer | potatoes, apples |
| O2 | ostrich | sweet potatoes, red beet |
| O3 | kangaroo | celery, pumpkin |
| O4 | insects | sweet potatoes, pea |
